# Regional COVID-19 measures and effects on subjective well-being in Germany: observing trends over time with data from a large population survey

**DOI:** 10.3389/fpubh.2025.1523691

**Published:** 2025-02-27

**Authors:** Emily Finne, Anna Christina Nowak, Oliver Razum

**Affiliations:** School of Public Health, Department of Epidemiology and International Public Health, Bielefeld University, Bielefeld, Germany

**Keywords:** COVID-19, subjective well-being, life satisfaction, non-pharmacological interventions, preventive measures, regional deprivation

## Abstract

**Background:**

COVID-19 measures in Germany varied during the pandemic, and it seems natural that in addition to factors such as incidence, health system capacity, etc., these interventions and their social and economic consequences had an impact on the evolution of the population’s well-being. Since the beginning of the pandemic, there has been a suspicion that the health burden would fall mainly on population groups with a lower socio-economic status, and that COVID-19, including the policy measures, could therefore contribute to increasing social inequalities in health. We examine several indicators of well-being over the course of the pandemic, analyze the effect of the stringency of the measures on subjective well-being and the extent to which certain social groups were particularly affected.

**Methods:**

Our analyses are based on 2020 and 2021 data from the German Socio-Economic Panel (SOEP), complemented by various regional indicators, including the COVID-19 measures. Data on subjective well-being during the pandemic phases were regressed on the phases, socio-demographic, economic and health-related indicators, stringency of measures and other regional indicators in multi-level models with the district as the top level. Up to *N* = 29,871 observations in 401 districts were included.

**Results:**

Overall, there was little decline in well-being up to the end of the observation period, and even some increase. When the effect of the stringency of the measures was taken into account, the changes were partially attenuated. However, stringency had little direct effect on well-being. People with disabilities and chronic pre-existing conditions were particularly affected by a reduction in well-being. In some cases, COVID-19 measures had slightly different effects in these groups.

**Conclusion:**

The effects of socio-economic indicators were not strong enough to suggest that lower social status is generally associated with a negative trend in well-being. According to our results, people with disabilities and chronic diseases, including severe obesity, should be given more attention in the future. A change in time-related outcomes when considering COVID-19 measures could indicate adjustment effects on well-being.

## 1 Introduction

In Germany, policy measures to contain the spread of COVID-19 (Non-Pharmaceutical Interventions, NPIs) were first introduced in March 2020, and the first “lockdown” started on 23 March 2020 with far-reaching restrictions on trade, freedom of travel, contact restrictions, and quarantine measures for infected persons. Another, longer lockdown was implemented late in 2020 until May 2021. In addition to the new situation with many uncertainties regarding health risk and economic consequences of the pandemic, some NPIs significantly restricted social and professional life, at least temporarily. Taken together, the pandemic situation resulted in stress with likely negative impact on the health and well-being of the population.

Already at the beginning of the pandemic it was suspected [e.g., (1)] that health burdens would primarily affect population groups with lower social status and could thus contribute to increasing social inequalities in health. These are seen as result of an unequal distribution of (material, psychosocial, behavioral, and sometimes biological) risks and resources in the population (2). Government measures can reduce or increase these inequalities. Besides preventing infection, NPIs in the context of the pandemic can be seen as a general stressor that requires resources to cope with, with lacking resources resulting in lowered health and well-being. Regionally varying policies like NPIs impact on different dimensions of the living context of residents and can affect social and health disparities by influencing locally available resources. Examining the effects of NPIs on the health determinants can provide further insights into how these measures have exacerbated health disparities.

Socially unequally distributed direct health effects of the COVID-19 pandemic have been shown internationally and nationally in various studies (3–5). People from socially deprived regions, for example, were more frequently affected by infections, severe disease progression and higher mortality rates (6–8). This also applied to people with a lower level of education (9), and to those with immigration or refugee experience (9–11). In Germany, higher incidences were found in socially better-off regions at the beginning of the pandemic, but these have reversed since the second pandemic wave at the end of 2020 to the disadvantage of socially deprived regions, where mortality was also higher (12, 13).

In addition, older people and people with pre-existing chronic health conditions, disabilities, and severe obesity are also more susceptible to infection and severe courses of the disease (14–16). Aside from that, they may depend on specific support in health and everyday life matters, which was shortened due to NPIs (17). In Germany, care, support, and work facilities for people with disabilities, for example, were closed during the lockdown (18). Due to contact restrictions, additional support needs arose for chronically ill or disabled persons, that could not be covered promptly by welfare support care services. Additional needs often had to be organized privately through family or neighborly support (19).

For the first year of the pandemic, our own analysis identified people with disabilities, chronic diseases, or obesity as those groups with the highest average decline in subjective health and well-being in Germany (20). This may have been due to the particular social isolation as well as greater psychological stress due to fears of infection and feelings of helplessness and abandonment. Overall, NPIs led to restrictions that were often particularly difficult for the chronically ill and disabled to cope with.

Subjective well-being is one important aspect of subjective health Although different conceptualizations and many different measures tapping into well-being exist, subjective well-being is widely accepted to comprise of three principal components (21): overall life satisfaction, which is defined as an overall assessment of one’s own life; satisfaction with important areas of life, such as work and health; and a predominantly positive emotional state with a low prevalence of negative affect. Affective well-being can be defined as the emotional evaluation of one’s own emotional state, whereby the ratio of positive to negative emotional states is decisive. The general and domain-specific forms of life satisfaction correspond to the cognitive-evaluative dimension of subjective well-being, defined by Diener et al. (22) as a cognitive evaluation of one’s own state of being. Subjective well-being is strongly correlated with mental health. Common mental disorders, such as anxiety and depression, are typified by predominantly negative affective states and are frequently associated with physical symptoms (23). Indicators of affective well-being, quality of life, and life satisfaction are also categorized as positive indicators of mental health (24). Subjective well-being is related to a variety of potential determinants at different levels as, for example, demographic and socio-economic indicators, health and functioning, personality, social support, culture, and regional infrastructure (21).

Multiple studies document losses in well-being and mental health during the pandemic. For example, respondents in a large British cohort study reported considerable stress due to life changes in the early days of the pandemic. Increased stress, in turn, was associated with a higher risk of anxiety and depressive symptoms (25). An international meta-review on changes in mental health during the pandemic concluded that the prevalence of possible depression or anxiety disorders increased by at least 0.2 standard deviations compared to the pre-pandemic period (26). Santomauro and colleagues arrived at an estimate of more than 25% global increases in prevalence for anxiety and major depression each within the first year of the pandemic. Regions with more strict restrictions on human mobility and those with high COVID-19 incidence rates showed the highest increases (27). Another international review also concludes that COVID-19-related restrictions were related to risk factors for mental illness in the general population, with female gender, low socio-economic status, young or old age and characteristics of the living environment proving to be risk factors for mental health problems (28).

For Germany, representative population data from the first months of the pandemic describes only small changes in subjective health and well-being indicators, while differences between social groups from the pre-pandemic remained stable (29, 30). Pandemic-specific survey data revealed an overall increase in depression and anxiety scores during the first COVID-19-wave, followed by a decline during the following months up to the beginning of 2021. Changes in life satisfaction were very small for the first wave but more marked for the second wave, while satisfaction with health even increased during the first wave and only slightly declined thereafter (31). Representative telephone surveys from before and during the pandemic also point to an overall trend toward a decline in mental health measures during the course of the pandemic, particularly in the long term (32).

Fewer studies examined the direct impact of contact restrictions and NPIs. A meta-analysis concludes that lockdown effects on mental health symptoms were small and heterogeneous whereas effects on positive psychological functioning could not be established (33). An Australian study showed more pronounced declines in mental health with lockdown, especially for mothers and those living in an urban environment (34). Aknin et al. (35) reveal associations between the stringency of national NPIs with measures of psychological distress and life evaluation across 15 countries in the period from April 2020 to June 2021. While an international review (36) came to the conclusion that the prevalence of depression was lower in countries with more stringent and early interventions to contain the virus, a meta-analysis showed an increased risk of mental health problems for those affected by isolation measures or quarantine (37). A longitudinal study over 2 years in the UK found a positive correlation between the stringency of NPIs and higher levels of depression and anxiety, especially during lockdown phases (38). Another study involving five European countries, including Germany, found NPIs stringency to be related to lower life satisfaction for 2020 (39). Overall, reported NPI effects on mental health and well-being measures are heterogeneous but seem predominantly negative. However, there are also indications that effects were unevenly distributed and larger in some social groups (31, 34), and differential consequences of NPIs are not sufficiently understood.

Altogether, different studies point to declines in mental health and well-being as consequence of COVID-19-related restrictions. After a first ‘shock’ at the beginning of the pandemic, adverse effects on mental health and well-being seem to evolve primarily in the medium- to long-term of the pandemic situation. Differences in outcomes in the international literature may be related to differences in pandemic exposure over time, the care situation, sociodemographic differences, cultural context and, not least, the type and severity of NPIs implemented. Evidence on factors explaining these developments is scarce and mostly indefinite (26).

We analyze trajectories of subjective well-being in Germany over the pandemic phases from the beginning in 2020 to early 2022 using data from a large household survey complemented by regional statistics on social indicators and implemented NPIs over time.

The main aim of the study was to investigate the extent to which the NPIs, with variations in their severity over time and regions, were negatively related to the subjective well-being of the German general population. To this end, we look at the extent to which well-being was affected differently in different social groups, whether NPIs explain temporal trends across the pandemic phases, and whether there were different effects of NPIs in different groups. We expect the stringency of regional government imposed NPIs to be associated with lower well-being over time. We also suppose that NPI stringency will explain parts of the temporal and regional differences in well-being. Furthermore, in line with the state of research on socially disadvantaged groups, we expect that the extent of the impact of more stringent NPIs depends on social characteristics (socio-economic status and sociodemographic characteristics as well as health limitations) and that marginalized groups are likely to be more affected. This implies statistical interactions between the severity of the NPIs and social indicators at the individual or regional level.

## 2 Materials and methods

### 2.1 Data base

The analysis is based on survey data from the German Socio-Economic Panel (SOEP) v.38.1 (40, 41), data compiled by infas, infas 360 and the IHPH Bonn as part of the ‘Corona Data Platform’ (now www.healthcare-datenplattform.de), and regional indicators from the INKAR database (Indicators and Maps on Spatial and Urban Development, www.inkar.de) of the Federal Institute for Research on Building, Urban Affairs and Spatial Development (BBSR).

The SOEP is a comprehensive, representative panel study conducted by the German Institute for Economic Research (DIW). It has been surveying the German population at both the household and individual levels on a range of economic, social and health-related indicators since 1984. Approximately 30,000 individuals from 15,000 households are surveyed on an annual basis. Households are allocated to regions according to district codes, which correspond to the NUTS3 regions defined by the EU classification system (42).

We used data from individuals with valid data during the pandemic and at least one survey wave before. We coded as baseline measurement (pre-Covid) the last measurement taken before the beginning of the pandemic. The data on outcomes relating to subjective well-being were derived from interviews conducted between 2020 and 2022, subsequent to the initial onset of the first wave of the SARS-CoV-2 pandemic in Germany.

Based on the date of the participant interview, the data were assigned to pandemic phases as categorized by the Robert Koch Institute (RKI) (43). Phases are shown in Table 1. The advent of the pandemic is marked by the start of the initial wave, which began in the tenth week of 2020 (phase 1). The first documented measures to contain the pandemic fall within this phase. In instances where individual details pertaining to the survey date were unavailable, a plausible value was employed wherever feasible. If the day in question was not provided, the 15th day of the month was assumed. Any missing survey years were replaced with the planned survey year, missing months were not replaced.

**Table 1:** Distribution of the SOEP interviews included in the analysis across the pandemic phases.

Phase	Period (KW)	Included observations
0: sporadic cases	5/2020–9/2020	1,539
1: First wave	10/2020–20/2020	8,777
2: Summer plateau 2020	21/2020–39/2020	3,677
3: Second wave (during which vaccines became available)	40/2020–8/2021	1,945
4: Third wave (VOC Alpha)	9/2021–23/2021	4,244
5: Summer plateau 2021	24/2021–30/2021	4,555
6a: Fourth wave (VOC Delta Summer)	31/2021–39/2021	3,134
6b: Fourth wave (VOC Delta autumn/winter)	40/2021–51/2021	1,946
7: Fifth wave (Omicron subl. BA.1 / BA.2)	52/2021–21/2022	54

The phasing follows the retrospective categorization of the RKI (43); VOC, Variant of Concern; KW, Calendar week.

Data were merged with data on regional characteristics. Regional information was retrieved from the Corona data platform and official sources (INKAR database). To comply with data protection regulations, this step and all analyses using regional identifiers were done on a secured computer terminal with a safe direct connection to the SOEP data base. Personal information was only exported aggregated over time and region, when sample sizes allowed. For district characteristics we used the values observed in 2019, representing the last complete pre-pandemic year. In the case of time-varying characteristics pertaining to COVID-19 and the NPIs (stringency levels, incidence rates, and the number of vaccinations administered), the data were initially aggregated at the weekly level and then added to the SOEP data set by calendar week and district code. The subsequent procedure is outlined in the following, where we also specify the used variables.

### 2.2 Measures

#### 2.2.1 Subjective well-being

All three components of subjective well-being (overall life satisfaction, domain-specific satisfaction, and affective well-being) are measured in the SOEP on a yearly basis in adults and operationalizations are shown in Table 2. For the purpose of this analysis as domain-specific life satisfaction only satisfaction with health is included.

**Table 2:** Measurement of subjective well-being in the SOEP.

Well-being aspect	SOEP survey question	Response options and range
Affective well-being (scale of 4 items)	“I’m going to read you a series of feelings. Please indicate how often or rarely you have experienced this feeling in the last 4 weeks. How often have you felt …?” … angry … worried … happy … sad	1 = very rarely to 5 = very often
General life satisfaction	“All in all, how satisfied are you with your life at the moment?”	From 0 = completely dissatisfied to 10 = completely satisfied
Satisfaction with own health	“How satisfied are you … … with your health?”	From 0 = completely dissatisfied to 10 = completely satisfied

The four items on affective well-being were aggregated into a scale by calculating the mean value. The items relating to negative affect were initially reversed so that higher values on the scale indicate a higher level of well-being. An examination of the retest reliability on a small sample after 4–7 weeks yielded a retest reliability of r_tt_ = 0.54 for this scale (44). Our own analysis yielded an internal consistency of *α* = 0.67 for the four items. General life satisfaction and satisfaction with health are each based on an 11-point answer scale. The evidence suggests that these one-item measures demonstrate good convergent and divergent validity (45). A test–retest reliability of r_tt_ = 0.66 was determined over a period of 1 year. Similarly, satisfaction with one’s own health achieved a one-year test–retest reliability of r_tt_ = 0.64 (46).

#### 2.2.2 Further covariates at person level

Additional variables derived from the SOEP on socio-demographic and socio-economic data are presented in Table 3.

**Table 3:** Other SOEP variables included at individual level.

Variable	Definition
Age	Calculated from year of survey and year of birth.
Gender (male)	Reference category: female.
Immigration history	To determine the immigration history, the SOEP combines various data based on the country of birth of the respondents and their parents. A first-generation immigration history is assumed for birth in the country of origin, a second-generation immigration background for birth in Germany and (grand-)parents with direct migration experience.
Marital status (married)	“What is your marital status?” Participants with the information “married” and “registered same-sex partnership” were classified as married.
Single parent	Binary variable extracted from variable on household type.
One-person household	Binary variable extracted from variable on household type.
Education	According to CASMIN classification: Information on school-leaving qualifications, classified according to the “Comparative Analysis of Social Mobility in Industrial Nations (CASMIN)” scheme; only the 3 main groups were differentiated (62).
Chronic health condition	“Have you been suffering from certain complaints or illnesses for at least a year or chronically?” Answer options: Yes/No
Disability	“Have you been officially recognized as disabled or severely disabled?” Answers: yes/no; if yes: free indication of degree of disability. A disability was classified as an officially recognized disability with a degree of 30% or more.
Overweight/obesity	Body height and weight were self-reported. The body mass index (BMI) was calculated from this as body weight in kg/(height in metres^2^). Values were then categorized as overweight for a BMI of 25 or more and as obesity for a BMI of 30 or more (63). The reference category is people with a BMI below 25.
Net equivalent income	The monthly net income is calculated from various household income data, whereby the number of persons is determined using modified equivalence weights. The per capita income in the household is calculated. A calculation rule can be found in (64).
Self-employment	“What is your current employment?” The answer option “Self-employed (including family members helping out)” was coded as self-employment.
Working hours per week	“(…) what is your average actual working time per week, including any overtime?” - Answer: free format.
Person in need of care living in household	“Is there anyone in your household who is permanently in need of help or care due to age, illness or disability?” Answers: Yes/no

#### 2.2.3 Regional indicators

##### 2.2.3.1 Regional deprivation

The values from 2019 of the revised form of the RKI’s German Index of Socioeconomic Deprivation (GISD) were used to record socio-economic deprivation at district level (47, 48). This index is intended to depict the socio-economic position of regions in relation to each other by weighting and summarizing district indicators on education, employment and income of the population as well as additional data from the Federal Employment Agency. The GISD score is standardized to values between 0 and 1, with higher values indicating greater deprivation. The index proved to be associated with lower life expectancy and higher cardiovascular and lung cancer mortality (47). Results from an earlier version indicated clear contextual effects beyond individual social status (49).

##### 2.2.3.2 Stringency index

In order to summarize the impact of regional NPIs over time, an index created by infas 360 was used, which summarizes the stringency of the prescribed COVID-19-related NPIs at district level on a daily basis.^1^ There is a total of 23 main categories of measures (including contact restrictions in public or in private space, school closures, mask requirements, travel and workplace restrictions among others), each with a different number of subcategories, which were recorded on a daily basis starting from 1st March 2020. The subcategories were ranked according to their severity, standardized, and summarized. The resulting index can theoretically assume values between 0 and 100. We aggregated stringency over time by computing the mean stringency value up to the beginning of the pandemic phase where the measurement took place, separately for each district. This was done to account for cumulative effects of stringency over time, since we did not expect people to react to changes in NPIs immediately or after a specific time period defined in advance.

To shed more light on the significance of certain NPI measures, we analyzed the intercorrelations of the days up to the measurement week in which each specific NPI was in force by a principal component analysis. Most of the NPIs were in force simultaneously, resulting in a single component representing most NPIs with loadings above 0.95, except of traveling restrictions, regulations at the work place, and home confinement. These were registered less commonly in the data base. We therefore refrained from analyzing separately specific measures but used the component score of the one component solution as alternative index for the severity of regional restrictions over time. The overall correlation between the stringency index and the component score in our sample was *r* = 0.828.

Additional data at the district level from the Corona Data Platform and from the INKAR database is described in Table 4. We selected indicators that are related to the COVID-19-related infection risk (number of cases, vaccinations, number of recovered, age of the population) or were directly relevant to care and support services (rurality, as population density is lower and infrastructure is poorer in rural areas, hospitals, childcare).

**Table 4:** Indicators used at district level.

Indicator	Source	Definition
German index of Socio-economic deprivation (GISD)	Michalski et al. (48)	Relative socio-economic position of district compared to other German districts; Weighted sum of indicators on education, employment, and income; standardized to 0–1 for lowest to highest regional deprivation.
Rurality	Regional planning data set (‘Raumordnung’)	Proportion of inhabitants in municipalities with a population density of less than 150 inhabitants per km^2^ (in %).
7-day incidence	Data set infections	Confirmed COVID-19 cases in the last 7 days per 100,000 inhabitants (as of 31 December 2020).
COVID-19 recoveries cumulated	Data set infections	Estimate of COVID-19 recoveries cumulated (by reporting date) - relative to the population in the district.
Vaccinations already administered cumulatively	Vaccination data set (original source RKI: see https://github.com/robert-koch-institut/COVID-19-Impfungen_in_Deutschland)	The data from the Corona data platform were employed (with daily updates). The preceding vaccinations (1st, 2nd and 3rd) up to the interview were aggregated so that cumulative values were available up to the date of the survey. These were relativized to the population figures for each district. Due to multiple vaccinations, the value achieved may exceed a proportion of 1.
Share of older people aged 65 and over	INKAR	Proportion of residents aged 65 and over in the population.
Average age	INKAR	Average age of the population.
Hospital care	INKAR	Index on hospital care.
Childcare rate for young children	INKAR	The proportion of children under the age of three who are enrolled in day care centers is expressed as a percentage of the total number of children in the corresponding age group.
Childcare rate for pre-school children	INKAR	The proportion of children aged between three and under 6 years old who are in day-care centers is expressed as a percentage of the total number of children in the corresponding age group.
Total population	INKAR	Total number of inhabitants.

Indicators in the upper part of the table were taken from different data sets of the Corona Data platform; INKAR, Indicators and Maps on Spatial and Urban Development (as of 31th Dec 2019); RKI, Robert Koch Institute.

### 2.3 Statistical analyses

All analyses were performed using R software (50), version 4.3.2 (v. 4.2.2 for regional analyses). To describe the data, means and standard deviations were calculated for continuous characteristics, and numbers of cases and percentages for categorical variables. To visualize the spatial distribution of stringency, aggregated data at district level were assigned to corresponding polygons via the NUTS3 code of the districts.

In order to ascertain the extent to which the pandemic, and NPIs in particular, have influenced the subjective well-being of the adult population, and the extent to which changes differ regionally according to social deprivation and other indices, multi-level linear regression models were created stepwise with well-being indicators as continuous dependent variables. At each level, the potential confounders were included based on statistical criteria (significance and model fit, as indicated by R^2^, AIC, and BIC), as the number of missing covariate values added up and inclusion of all values would likely have led to further case losses. As our principal objective was to ascertain the explanatory value of NPIs in specific risk groups, the relevant variables were included irrespective of model fit. Interaction terms were tested individually and only retained in the model if they were significant, in order to avoid overfitting.

In the first stage of the analysis, intraclass correlations (ICC) were calculated for the null models to quantify the cluster effect at the district level. Subsequently, a model comprising indicators at the individual level and a random intercept for the district was identified. To examine changes in well-being between the current measurement and the pre-pandemic baseline, the baseline was controlled for as a covariate [ANCOVA approach; see, for example (51)]. In order to map the course of the values over time, the pandemic phase was taken into account as a predictor at this level, with the inclusion of only statistically significant individual indicators. Subsequently, district level control variables were incorporated and interactions with the stringency of NPIs were evaluated. In a subsequent step, random slopes for the phases were permitted.

In addition to the potential clustering at the district level, there is a further dependency of the values within individuals. The dependencies within individuals, as indicated by the ICC values, were largely accounted for by the inclusion of pre-pandemic well-being scores, and three-level models lead to convergence issues in some cases. Accordingly, no additional, third level of analysis was considered, and only random effects at the district level were included.

The spatial autocorrelation of the residuals aggregated at the district level was evaluated using Moran’s I, a test for unexplained spatial dependencies, for the resulting final models.

The SOEP is conceptualized as a representative survey of the population of Germany, with the appropriate case weights provided for each wave. However, weights are not intended to yield representative data at the district level or for short time intervals. Consequently, we do not claim representativeness at that level and rely on unweighted statistics. As a sensitivity analysis, we reassess the model results with weighted data (see Supplementary Table S4).

## 3 Results

### 3.1 Descriptive analyses

Table 5 describes the SOEP cases included in the subsequent analyses for the two SOEP survey waves 2020 and 2021. The case numbers refer to the cases included in the regression models for the outcome ‘satisfaction with health’ (*N* = 15,938 from 2020 and 13,933 from 2021). The number of valid cases was slightly lower for the other two outcomes. Weighted statistics can be found in the Supplementary Table S1.

**Table 5:** Description of the sample.

Survey wave ➔	2020					2021				
	M	Md	SD	range	*N*	M	Md	SD	range	*N*
Outcomes and initial baseline values before the pandemic										
Affective well-being	3.599	3.75	0.692	1–5	14,149	3.718	3.75	0.687	1–5	13,922
Initial value affective well-being	3.691	3.75	0.678	1–5	14,152	3.635	3.75	0.686	1–5	13,933
Life satisfaction	7.546	8.0	1.630	0–10	15,912	7.547	8.0	1.615	0–10	13,904
Initial value life satisfaction	7.529	8.0	1.662	0–10	15,935	7.614	8.0	1.575	0–10	13,931
Satisfaction with health	6.889	7.0	2.166	0–10	15,938	7.017	7.0	1.993	0–10	13,933
Initial value health satisfaction	6.900	7.0	2.177	0–10	15,938	6.819	7.0	2.103	0–10	13,933
Covariates at individual level (last measurement before the pandemic)										
Age in years	48.237	48.0	16.819	18–99	15,938	51.884	53.0	16.871	19–100	13,933
Net equivalent income in EUR	1927.345	1697.5	1799.300	23.3–133333.3	15,938	2362.0	2000.0	2876.513	0–133333.3	13,933
Working hours/week	36.800	40.0	10.793	0–80	15,667	37.20	40.0	10.958	0–80	13,705
Number of children up to 13 years	0.614	0	1.038	0–8	15,938	0.422	0	0.799	08	13,933
Categorical and dummy variables										
	*N*		Percent			*N*		Percent		
Gender (male)	7,669		48.117%			6,533		46.888%		
Education (Casmin 3-stage)										
low	4,852		31.394%			3,331		24.462%		
medium	6,449		41.728%			5,884		43.211%		
high	4,154		26.878%			4,402		32.327%		
Immigration history										
None	10,795		67.731%			11,380		81.677%		
Second generation	923		5.791%			708		5.0815%		
First generation	4,220		26.477%			1845		13.242%		
Self-employed	813		5.101%			1,092		7.838%		
Single parent	1,448		9.085%			1,137		8.160%		
One-person household	2,517		15.792%			2,593		16.610%		
Married	9,000		57.629%			7,955		57.276%		
Disability (degree > = 30%)	1,468		9.211%			1,619		11.620%		
Chronic illness	5,652		35.293%			5,921		42.496%		
	*N*		Percent			*N*		Percent		
Overweight	5,520		34.634%			5,039		36.166%		
Obesity	3,296		20.68%			2,936		21.072%		
Last survey year pre-pandemic (for baseline measures)										
2020	1,628		10.215%			3,860		27.704%		
2019	14,307		89.767%			10,072		72.289%		
2018	3		0.02%			0		3.54%		
2017	0		0%			0		0.33%		
2016	0		0%			1		0.05%		

M, mean; Md, median; SD, standard deviation; range = maximum - minimum; *N*, number of valid cases (in analyses).

The values from the last survey prior to the pandemic are shown as the initial values. The year of the survey at the bottom of the table shows that these values are mostly from surveys conducted in 2019, and in some cases from early 2020. Only very rarely were baseline values collected further back. All covariate values are also based on pre-pandemic measurements. On average, there are very small changes in the measured outcomes between the two survey waves. The independent variables are also very similarly distributed in both waves. Weighted outcome values were similar to unweighted values, especially in 2020 (Supplementary Table S1). The weighting affected the distribution of some socio-demographic indicators, which were all controlled for in our main analyses.

Table 6 presents descriptive statistics for the potential predictors collected at district level. Characteristics that changed over time were averaged at the district level. Changes in the three outcomes averaged at district-level are described at the bottom of Table 6.

**Table 6:** Included district indicators.

	M	Md	SD	Range
GISD score	0.55	0.56	0.17	0–1
Total population	207,398	154,899	245,162	34,193–3,669,491
Rurality	29.5	21.9	30.1	0–100
Share of older people (aged 65 and over) %	22.6	22.2	2.96	15.6–32.7
Average age	44.7	44.5	2.03	39.9–50.5
Index hospital supply	6.29	5.48	3.88	0–28.7
Childcare rate for young children	34.7	31.3	12.6	14.4–65.8
Childcare rate for pre-school children	90.3	90.8	3.98	72.6–100
Varying in time:				
Stringency	35.8	35.4	6.93	14.2–55.9
Average stringency until survey date	29.8	29.8	4.57	15.2–42.7
7-day incidence (per 100 k)	49.2	42.4	28.3	0–198
Cum. Proportion recovered	0.02	0.02	0.01	0–0.06
Cum. Proportion of vaccinations (total, with repeat)	0.16	0.15	0.12	0–0.69
Percentage changes in outcome aggregated at district level				
Affective well-being	−0.10	−0.17	2.21	−8.16–15.0
Life satisfaction	−0.37	−0.29	2.68	−20–14.0
Satisfaction with health	0.74	0.78	3.74	−30–20.2

M, mean; Md, median; SD, standard deviation; range = maximum - minimum; GISD, German Index of Socioeconomic Deprivation.

**Figure 1:**
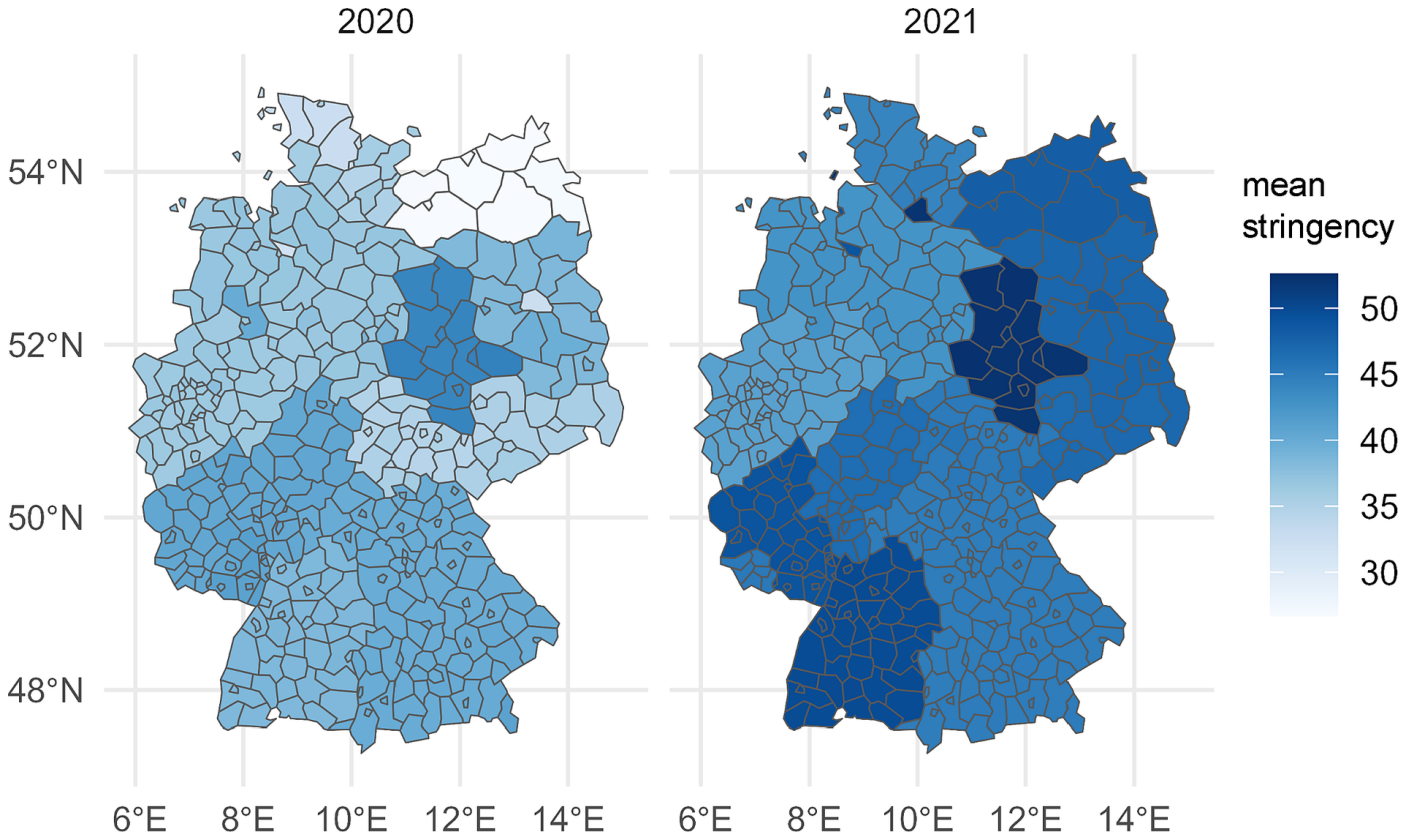
Average values of the stringency index for the severity of COVID-19 measures in Germany at district level (own presentation of infas 360 data from www.healthcare-datenplattform.de).

The following maps (Figure 1) show the mean values of the stringency index at district level for the survey waves 2020 and 2021. It is clear that the average stringency of measures was higher in 2021. It is also clear that there is less variation in stringency between districts than between the larger federal states.

The analyses of changes in well-being and NPIs refer to the pandemic phases as retrospectively classified by the RKI. The phases, their meaning and duration, as well as the corresponding case numbers (again, valid cases from the health satisfaction model) are shown in Table 1. Phase 0 serves as the time reference category of the dummy-coded variable in the regression models. Although the number of cases for the last included phase 7 is very small, we did not combine it with the previous phase as this would have resulted in a very long time period. However, the results for phase 7 should be interpreted with caution.

### 3.2 Results of multi-level models

The models were calculated separately for each of the three outcomes with the individual data nested in the districts as the upper level. The ICCs (see Supplementary Table S2) at district level for the three outcomes fall within the range of 0.007–0.015 and can therefore be regarded as low with a proportion of explained variance of less than 2%. This means that the outcomes are only slightly spatially clustered at the district level. ICCs for the individual level ranged from 0.492 to 0.574, typical for repeated measures from the same persons. However, there was no variance at person level left after adjusting for individual baseline scores, so that no person random effect was included.

Mainly two models were compared for every well-being outcome. First, a model containing only the pandemic phases and significant sociodemographic individual-level covariates was computed. This model served as benchmark for assessing regional stringency effects. Phases were adjusted for variables that may have acted as confounders mainly to adjust for possible differences in sample characteristics between the phases. Second, the complete model consists of the predictors of interest and significant control variables at individual and district level, particularly including NPI stringency and other COVID-19-related measures. Interaction terms between stringency and sociodemographic indicators at individual and district level were included when significant. A model with a random-slope for the pandemic phase was also tested but this model failed for all outcomes because of convergence problems due to singularity.

The final models (Table 7) thus include predictors at individual and district level as well as significant interactions with the stringency index. Residuals from these models were tested for unexplained spatial dependencies, but Moran’s tests indicated no meaningful spatial autocorrelations (Supplementary Table S3).

**Table 7:** Coefficients (standard errors) of the final models for the three outcomes.

	Affective well-being		Life satisfaction		Health satisfaction	
	Only phase (adjusted)	Complete model	Only phase (adjusted)	Complete model	Only phase (adjusted)	Complete model
Number of observations	*N* = 27,409 in 400 districts		*N* = 29,464 in 401 districts		*N* = 29,871 in 401 districts	
Fixed effects:						
Intercept	1.632 (0.025)***	1.498 (0.030)***	3.758 (0.059)***	3.659 (0.072)***	3.523 (0.056) ***	3.737 (0.071) ***
Pre-pandemic score	0.555 (0.005)***	0.555 (0.005)***	0.507 (0.005)***	0.505 (0.005)***	0.502 (0.005) ***	0.493 (0.005) ***
Pandemic phase (ref. = 0)						
1	−0.114 (0.016)***	−0.051 (0.017)**	−0.057 (0.038)	−0.033 (0.040)	0.025 (0.045)	−0.010 (0.048)
2	−0.074 (0.018)***	0.036 (0.024)	−0.037 (0.042)	0.017 (0.058)	0.003 (0.050)	−0.066 (0.069)
3	−0.072 (0.026)**	0.067 (0.031)*	0.122 (0.050)*	0.204 (0.065)**	0.175 (0.058)**	0.102 (0.0751)
4	0.083 (0.017)***	0.244 (0.028)***	0.010 (0.041)	0.091 (0.065)	0.400 (0.048)***	0.318 (0.077)***
5	0.058 (0.017)***	0.216 (0.028)***	−0.090 (0.0402) *	−0.022 (0.064)	0.306 (0.048)***	0.220 (0.076) **
6a	0.075 (0.018)***	0.241 (0.028)***	−0.066 (0.043)	0.015 (0.065)	0.193 (0.051)***	0.108 (0.077)
6b	−0.049 (0.019)*	0.145 (0.030)***	−0.225 (0.0467) ***	−0.092 (0.072)	0.001 (0.055)	−0.080 (0.0799)
7	−0.154 (0.080)^#^	0.087 (0.085)	−0.503 (0.191) **	−0.311 (0.203)	−0.081 (0.223)	−0.161 (0.230)
Medium stringency of previous NPIs (increment of 5)	-	−0.008 (<0.001)**	-	−0.008 (<0.001)	-	0.002 (0.001)
GISD score 2019	-	−0.002 (0.024)	-	0.139 (0.058)*	-	0.143 (0.067) *
Control variables individual level (pre-pandemic score)						
Disability	−0.049 (0.012)***	−0.049 (0.012)***	−0.184 (0.028) ***	−0.169 (0.028)***	−0.497 (0.033)***	−0.464 (0.033)***
Chronic illness	−0.065 (0.008)***	−0.065 (0.008)***	−0.156 (0.018)***	−0.154 (0.018)***	−0.438 (0.022)***	−0.417 (0.022)***
Overweight	-	−0.003 (0.008)	-	0.001 (0.019)	-	−0.096 (0.022)***
Obesity	−0.024 (0.008)**	−0.026 (0.009)**	−0.062 (0.020)**	−0.060 (0.022)**	-	−0.323 (0.026)***
Age (5-year increment)	0.006 (<0.001)***	0.005 (<0.001)***	−0.044 (<0.001) **	0.015 (<0.001)***	−0.060 (<0.001)***	−0.056 (<0.001) ***
Age^2^	-	-	0.003 (<0.001)***	0.003 (<0.001)***	-	-
Gender (male)	0.092 (0.007)***	0.092 (0.007)***	−0.057 (0.016)***	−0.058 (0.016)***	0.061 (0.019)**	0.078 (0.019)***
Education (Casmin 3) (ref = low)						
Medium	0.014 (0.009)***	0.012 (0.009)	-	-	-	-
High	0.024 (0.010)*	0.025 (0.010)*	-	-	-	-
Immigration history (ref. = none)						
Parents/ s generation	0.007 (0.015)	0.011 (0.015)	0.064 (0.037)^#^	0.066 (0.037)^#^	0.031 (0.043)	0.044 (0.043)
Self /first generation	−0.036 (0.010)***	−0.028 (0.010)**	0.088 (0.023)***	0.095 (0.023)***	0.104 (0.026)***	0.114 (0.026)***
Log of net equivalent income	0.040 (0.007)***	0.044 (0.007)***	0.165 (0.016)***	0.169 (0.016)***	0.209 (0.018)***	0.188 (0.018)***
Single parent	-	-	−0.089 (0.031)**	−0.085 (0.031)**	-	-
Number of children up to 13 years	-	-	0.041 (0.010)***	0.043 (0.010)***	-	-
Married	-	-	0.098 (0.020)***	0.102 (0.020)***	-	-
Person in need of care in household	-	-	−0.195 (0.040)***	−0.197 (0.040)***	−0.232 (0.046) ***	−0.234 (0.046)***
Control variables at district level						
7-day incidence (increment of 10)	-	−0.007 (<0.001)***	-	−0.003 (<0.001)**	-	-
Cum. Number of vaccinations	-	−0.055 (0.017)***	-		-	-
Childcare rate for pre-school children (2019)	-		-	−0.009 (0.003)***	-	-
Interactions with stringency						
Stringency x incidence	-	0.002 (<0.001)***	-	-	-	-
Stringency x disability	-	−0.007 (<0.001)*	-	-	-	-
Stringency x chronic illness	-	0.005 (<0.001)*	-	-	-	-
Stringency x GISD	-	-	-	-	0.006 (0.003)^#^	
Stringency x rate care preschool	-	-	-	−0.002 (<0.001)**	-	-
Random effects (standard deviations)						
Districts	0.030	0.029	0.094	0.081	0.097	0.096
Error	0.555	0.554	1.353	1.352	1.610	1.604

# *p* ≤ 0.10, * *p* ≤ 0.05, ** *p* ≤ 0.01, *** *p* ≤ 0.001; All continuous predictors were grand-mean centered; for binary variables, the non-occurrence is coded 0 as reference category.

All coefficients are adjusted for the pre-pandemic level of the outcome value. This means that, in fact, the change compared to this baseline adjusted for the initial score is analyzed, and coefficients reflect that change. To be more precise, the coefficients relate to the expected change in well-being when a predictor changes by one unit, on condition that the baseline well-being score is held constant. The coefficients for the phases furthermore relate to changes in well-being compared with phase 0, assuming a constant baseline value. That means we look at well-being changes relative to phase 0.

Looking at the changes in affective well-being over the pandemic phases, adjusted only for sociodemographic indicators and pre-pandemic well-being, there was a significant decline during the first phase with lowered values during phases 2 and 3 followed by slightly increased values beginning with phase 4 (which started during the second lockdown) and a decreasing trend starting with phase 6b in autumn/winter 2021.

The complete model shows only a small negative effect of preceding regional NPI stringency on affective well-being, as well as negative effects of incidence rate and vaccination coverage. However, there were some significant interaction effects with stringency involved: With higher stringency 7-day-incidence had a smaller negative effect. Furthermore, with higher stringency the disadvantage of people with disabilities was larger while that of people with chronic diseases was less pronounced.

Effects of the pandemic phase on affective well-being changed when accounting for NPIs and other regional COVID-19-related indicators. However, other than we expected it was not only that stringency of NPIs explained some of the declines in affective well-being, but adjusted for stringency as well as incidence and vaccination rate, affective well-being only showed a very small decline in phase 1 and then was even increased, especially from phase 4 on.

Overall, life satisfaction showed a slight increase in phase 3 and a small decrease in phase 5 and then seems to start to decline more manifestly with phase 6b in autumn/winter 2021. Again, people with disabilities or chronic diseases as well as those with obesity stated a significantly lower life satisfaction. When district level predictors were included, stringency showed no significant effect but higher 7-day-incidence was associated with a significantly lower life satisfaction. A higher regional rate of care for preschool children was related to a life satisfaction decline, and this effect was more pronounced with more stringent NPIs.

Controlling for stringency and other regional variables, the observed increase in life satisfaction in phase 3 was more marked. All other phase coefficients were no longer significant although predicted values remained higher with stringency adjusted. That is, in this case, all declines in life satisfaction could be explained by NPIs and other regional indicators.

Satisfaction with own health showed increases from phase 3 (second wave including lockdown) to phase 6a (summer 2021) but showed no declines compared to the beginning. Controlling for stringency and regional deprivation, these positive effects were slightly reduced and only remained significant for phase 4 and 5 (early to mid-2021). Stringency itself showed no significant main effect, but health satisfaction was higher in more deprived regions, more so when stringency was high.

The expected negative effect of NPI stringency could only be approved for affective well-being, where it was more pronounced for people with disabilities and lower for those with chronic diseases. Regional deprivation only proved to be statistically significant for life satisfaction and health satisfaction, but contrary to expectations, higher deprivation was more likely to be associated with improvements. Individual as well as district characteristics in connection with family life (single parent, married, number of children, childcare ratio) were only significantly associated with general life satisfaction. With regard to satisfaction with health, previous health problems in the form of chronic illnesses, obesity and disabilities played the most significant role. For both measures, a person with need of care living in the household was associated with lower satisfaction.

Not all of the classic socio-economic characteristics displayed associations with well-being. While income was positively related to all outcomes, education played a minor role, and a personal immigration experience was associated with a slightly lowered affective well-being but higher satisfaction with health and life in general. Particularly, there were no differential effects of stringency identified for these characteristics that would point to more negative effects in socio-economic weak groups. Besides the GISD score most other regional indicators did not explain additional variance. Altogether, the analyses reveal people with chronic conditions (including obesity) and disabilities as those in all aspects of well-being most negatively impacted by the overall pandemic situation, with stringency directly related only to affective well-being.

We compared our results to a model where we used the principal component score of NPIs instead of stringency, and found very similar results (Supplementary Table S4). We also compared our results to those from models using individual weights for SOEP 2020 and 2021 participants (Supplementary Table S4). There are some differences in the phase coefficients for affective well-being and life satisfaction. However, these would not dispute our conclusions.

## 4 Discussion

The COVID-19 pandemic was accompanied by significant social and economic disruption. In addition to the direct health consequences of illness and death, the pandemic containment measures (NPIs) posed a challenge to the population. The aim of this study was to investigate the extent to which these NPIs, in their regionally varying severity, were negatively associated with the subjective well-being of the general population in Germany over time, the extent to which the well-being of different social groups was affected during the pandemic, whether variations in NPIs can explain temporal trends, and to what extent different effects of NPIs can be demonstrated for different social groups.

### 4.1 Well-being trajectories

Observed changes in affective well-being and life satisfaction during the pandemic phases are consistent with results reported in the mental health literature, which show an initial small deterioration and then, after a brief improvement, indicate a long-term deterioration (32, 52). Our findings are also similar to those of Hettich et al. (31), which were based on a SOEP sub-sample but used different measures of affective well-being (depression and anxiety scales). There were, however, heterogeneous results, and even within Germany conclusions differed. Overall, in line with the literature, changes in positive well-being indicators were small and also smaller compared to symptoms of stress, depression or anxiety (26, 33, 53).

In Germany COVID-19 vaccines were distributed from the end of 2020, beginning with prioritized risk groups (older age specific chronic illness, and disability at the second prioritization level). It is therefore likely that initial uncertainties and fears of COVID-19 infection, which are related to mental well-being (54), explain some of the declines during early phases (before vaccine availability). They may also have been an influencing factor in later phases, when it got obvious that vaccines do not completely prevent from infections, and the second wave with high infection rates was encountered. Since NPIs left large parts of well-being changes unexplained, uncertainty and fears which were not measured in our study are likely influences.

The small changes in well-being can be interpreted in line with resilience research. There are different typical trajectories of psychological functioning in the face of disease outbreaks and natural disasters, with resilience (stable mental health in the face of severe stressors) being the most common (55). This was even the case among people hospitalized with SARS in 2003 (56). Thus, adapting to stressors and maintaining or restoring good mental health is the dominant response in crisis situations. Individual differences in well-being trajectories are not clearly identifiable when looking at group means but may depend on more individual constellations of risks and resources (55). We plan to further examine such resilience factors in the future.

### 4.2 Relationships between NPIs and well-being

Although the stringency of NPIs varied with the pandemic phases, the latter were much more strongly associated with well-being than the severity of NPI-related restrictions when both were included in the model. A negative main effect of stringency on well-being could only be shown for affective well-being, but the effect was rather small (for every 1 SD increase in stringency, affective well-being decreased by about 0.02 SDs overall). This result aligns with the conclusion of a meta-analytic review of lockdown effects which could not establish effects on positive psychological functioning (33). Nevertheless, the inclusion of NPIs and other COVID-19-related variables explained some of the variation in well-being across the pandemic phases. More positive developments over the course of the pandemic were observed for affective well-being and life satisfaction when stringency and variables such as number of vaccinations or district-level incidence were held constant, while positive developments in health satisfaction were partly explained by these COVID-19-related variables.

The relative stability of well-being measures such as life satisfaction or affective well-being is thought to be a consequence of adapting internal standards for evaluating one’s life or feelings to changing situations and environments. In terms of the pandemic, Schmidtke and colleagues (53) came to the similar conclusion that mental well-being and especially life satisfaction of German workers adapted to the pandemic within months.

Our result that the inclusion of COVID-19-related variables as NPIs resulted in less negative or even significantly positive changes, might therefore be interpreted as indication of these adaptive processes in well-being standards. That is, well-being was adjusted upwards, which only became visible when stringency effects were held constant. This would also mean that, without these adaptions, changes during the pandemic would have been clearly more negative. For health satisfaction, the reduction of positive effects over time when adjusting for stringency could also be interpreted as neutralizing parts of an upwards adjustment. Health satisfaction may have increased during the pandemic because mainly healthy people compared their health status to that what could have happened or to the health of people with severe course of COVID-19 who were very present in the media even when infection rates were low in Germany. This effect might have been reduced when factoring in negative effects of NPI stringency.

### 4.3 Effects on well-being in different social groups

The relationships with socio-economic indicators were not as consistent and strong as to conclude that lower SES was generally associated with negative developments in well-being during the pandemic, as emphasized by models of health inequalities, although higher income was in fact constantly related to better well-being in all three indicators. Instead of those with low socio-economic status, overall, people with disabilities and chronic illnesses were revealed as the most affected groups.

Our hypotheses regarding differential effects of NPI stringency in different social group were also only partially confirmed. We could not reproduce interactions with stringency for socio-economic indicators which were reported before (39). There were some differential effects of stringency but these effects were also hardly dependent on socio-economic status, but again rather on the previous burden of disease or disability. In line with models of health inequality (2), this can be explained by the greater vulnerability to health outcomes and a particular loss of resources in these groups.

According to our results, people with disabilities, chronic diseases and severe obesity should receive more attention in the future, especially with regard to the differential effects of NPIs. People with chronic diseases and, in particular, people with disabilities have received little consideration in the COVID-19 literature compared with other marginalized groups (57). For both groups there was a distinct deterioration in affective well-being, general life satisfaction and, in particular, health satisfaction. This confirms the results of our own earlier analysis, which showed that in the SOEP 2020 wave, declines in indicators of subjective health were predominantly observed in groups of people with pre-existing health problems, but that these were by no means homogeneous risk groups (20). Uncertainties and fears of infection likely play a role in these groups (18, 58). The results also point to problems in healthcare provision and utilization. While NPIs aimed at reducing infection risks especially for vulnerable groups including chronic ill and disabled persons, these groups also faced specific hardships. Coping with everyday life was made more difficult for example by restrictions on support services (17). Access to information was often hampered by the lack of non-discriminatory services, and discrimination in the health care system was also reported (59).

Furthermore, our results on life satisfaction also include indications of a higher pandemic burden on informal caregivers who care for others at home which may also be due to restrictions in outpatient care services (19).

Interestingly, the interaction between disability and NPI-stringency differed from that between chronic disease and stringency. While the interaction between disability and stringency indicated a stronger negative effect of stringency on affective well-being for disabled people, a less negative effect of stringency was found for chronically ill people. The scarce literature shows that in Germany, young adults with chronic illnesses reported the NPIs in a qualitative study as a largely positive experience and adhered strictly to the measures to protect their own health. In addition, the move to online formats in education and work was highly valued, as it had a positive impact on opportunities for social participation (58).

People with disabilities, on the other hand, experienced further limitations in their daily lives due to the lack of personal assistance, lack of health services, or difficulties in accessing vaccines (17, 18, 60). It became clear that restrictive measures particularly with regard to social participation should be further examined (61).

In this context, it would be particularly important to investigate which individual-level risk and protective factors determine the extent to which the well-being and mental health of these groups improve or deteriorate, and which factors mitigate the negative effects of measures such as contact restrictions.

It can be concluded that the policy in Germany of prioritizing certain vulnerable groups, e.g., in terms of vaccination protection, was justified, although obviously not sufficient.

The conflict between protection from infections and negative consequences of contact restrictions, as well as reductions in health and care services has to be approached with the inclusion of people with disabilities and chronic illnesses or their representatives when planning future responses to pandemics or other crises. In Germany, such efforts have been addressed, for example, by the German Association for Rehabilitation (DVfR), who systematically reappraised experiences from the first year of the pandemic and derived recommendations for action and research needs (61).

Considering demographic changes with a rapidly aging population and associated increases in chronic diseases and need for care, the pandemic situation accentuated constrictions in the German care system that have been obvious and under discussion for some time [e.g., (19)]. The impact of the pandemic on social participation is a particularly important aspect of this (61). More people are likely to be dependent on support in the future, so that a network of support and information services for future pandemics and other crises should be established.

### 4.4 Strength and limitations

To our knowledge, this is the first study to analyze changes in well-being in relation to regional NPI stringency over time for the German general population. The use of a large longitudinal population survey with established instruments is a strength and allowed us to include pre-pandemic measures of personal and regional characteristics. It also allowed the addition of official regional statistics and COVID-19-related data with high temporal and regional resolution. However, methodological limitations affecting our results cannot be excluded.

First, with regard to the pandemic phases, it should be noted that different people were interviewed in each phase, so that no intra-individual longitudinal changes could be analyzed over the phases. We adjusted our analyses for many potentially relevant covariates, but we cannot exclude the possibility that temporal changes over the pandemic phases are biased due to differences in sample composition. We therefore repeated our analysis using the individual survey weights for 2020 and 2021 with our final models, which resulted in slightly different estimates of changes in well-being over time. However, other coefficient estimates (particularly those for the identified risk groups such as people with disabilities) as well as interactions with stringency remained almost unchanged. It should be also emphasized at this point, that institutionalized people who were most affected by NPIs [e.g., (15)] were not sufficiently represented in this study, as the sampling scheme is based on private households.

Second, although the results were adjusted for the pre-pandemic values of the same people, which results in the analysis of longitudinal changes, we cannot draw causal conclusions from the analyses. We are looking at individual changes over time in relation to COVID-19-related changes over time, controlled for covariates, but obviously the pandemic conditions are not the only events or circumstances that have changed during the course of the pandemic that may have affected well-being.

Finally, the stringency index we used is a plausible summary of the NPIs, but it was inevitably an *ad hoc* construction without a theoretical basis. The extent to which weighting individual NPIs to produce an overall score reflects the actual (perceived) constraints faced by the population has not been investigated. We planned to examine individual NPIs in addition to stringency, but found that they were highly correlated and could essentially be summarized by only one principal component. This component score was used in additional analyses, and with the exception of the trajectory of health satisfaction in phases 4 and 5 only, the results were very similar to those of our main analyses. We therefore conclude that, with the exception of the exact pattern of change across phases, our results can be considered robust in terms of measuring NPI intensity and representativeness.

However, another issue may be the way in which we have aggregated NPIs that were in place before the actual measurement of well-being. Our assumption was that well-being would not change immediately after short-term changes in NPIs, but that COVID-19-related burdens would accumulate over time in their impact on well-being. This assumption might be incorrect and does not sufficiently take into account the adjustment processes in well-being that take place over time. The temporal processes in response to the pandemic should be further investigated, as well as the factors that buffered or amplified pandemic stress in different groups.

## 5 Conclusion

In the future, it will be particularly important to identify the key risk and protective factors that determine resilience to the limitations imposed by the pandemic. Links have already been identified, for example with different patterns of psychological coping (52). Future research should focus on groups with pre-existing health conditions and disabilities. Household assistance was often omitted due to contact restrictions and infections. Households with persons in need of care also demonstrated comparably great declines in well-being and as such should be kept in mind in future surveys. Especially institutionalized people are underrepresented in population surveys, and it must be prevented that they are left behind in future crisis situations. In the light of other studies, it also seems important to continue to monitor the development of mental health in the population over the long term in order to identify support needs for those who lack the necessary resources for resilience.

## Data Availability

The data analyzed in this study is subject to the following licenses/ restrictions: the data sets from Socio-economic Panel (SOEP) are held by the DIW Berlin. The scientific use file of the German Socio- Economic Panel is made available free of charge to universities and research institutes for research and teaching purposes when signing a data distribution contract with the DIW. Requests to access these datasets should be directed to soepmail@diw.de. Other datasets on COVID- 19-related indicators and regional indicators are freely available from the sources given in the text.

## Footnotes

^1^ https://www.infas.de/corona-datenplattform-publikationen-und-open-data
