# Supplementary material for: Regional COVID-19 measures and effects on subjective well-being in Germany: observing trends over time with data from a large population survey
Source: Front Public Health. 2025 Feb 27;13:1523691. doi: 10.3389/fpubh.2025.1523691 (PMC11905229; doi:10.3389/fpubh.2025.1523691)
Supplement: Supplementary file 1 [file Table_1.docx]

Supplementary Material

# Table S1: Descriptive statistics of the weighted sample

| Survey wave 🡪 | 2020 | | | 2021 | | |
| --- | --- | --- | --- | --- | --- | --- |
|  | M | Md | SD | M | Md | SD |
| *Outcomes and initial baseline values before the pandemic* | | | | | | |
| Affective well-being | 3.592 | 3.75 | 0.697 | 3.704 | 3.75 | 0.684 |
| Initial value affective well-being | 3.681 | 3.75 | 0.681 | 3.616 | 3.75 | 0.696 |
| Life satisfaction | 7.469 | 8.0 | 1.633 | 7.448 | 8.0 | 1.672 |
| Initial value life satisfaction | 7.477 | 8.0 | 1.662 | 7.518 | 8.0 | 1.608 |
| Satisfaction with health | 6.699 | 7.0 | 2.155 | 6.905 | 7.0 | 2.027 |
| Initial value health satisfaction | 6.699 | 7.0 | 2.171 | 6.661 | 7.0 | 2.166 |
| *Covariates at individual level (last measurement before the pandemic)* | | | | | | |
| Age in years | 50.693 | 51.0 | 18.015 | 52.653 | 54.0 | 18.124 |
| Net equivalent income in EUR | 2074.029 | 1866.67 | 1646.031 | 2156.745 | 2000.0 | 1446.06 |
| Working hours/week | 37.348 | 40.0 | 10.476 | 37.405 | 40.0 | 10.258 |
| Number of children up to 13 years | 0.319 | 0 | 0.717 | 0.281 | 0 | 0.658 |
| *Categorical and dummy variables* | | | | | | |
|  | Weighted Percent | | | Weighted Percent | | |
| Gender (male) | 48.67 % | | | 49.16 % | | |
| Education (CASMIN 3-stage) | | | | | | |
| low | 32.05 % | | | 30.70 % | | |
| medium | 42.90 % | | | 41.94 % | | |
| high | 25.05 % | | | 27.36 % | | |
| Immigration history | | | | | | |
| None | 78.26 % | | | 81.99 % | | |
| Second generation | 5.65 % | | | 4.70 % | | |
| First generation | 16.09 % | | | 13.32 % | | |
| Self-employed | 4.38 % | | | 4.85 % | | |
| Single parent | 7.06 % | | | 6.48 % | | |
| One-person household | 23.94 % | | | 27.20 % | | |
| Married | 50.83 % | | | 50.02 % | | |
| Disability (degree >= 30%) | 11.53 % | | | 13.86 % | | |
| Chronic illness | 40.00 % | | | 42.86 % | | |
| Overweight | 35.28 % | | | 36.93 % | | |
| Obesity | 20.76 % | | | 21.23 % | | |

# *Note*: M = mean, Md = median, SD = standard deviation; ranges and number of valid cases are the same as in table 5 in main text; SOEP individual weights for 2020 and 2021 were used, respectively

# Table S2: Intraclass correlations (ICCs):

| Outcome | | Cluster: district | Cluster: person in district |
| --- | --- | --- | --- |
| Affective well-being | empty model | 0.010 | 0.541 |
|  | adjusted for baseline score | <0.001 | n.a.* |
| Life satisfaction | empty model | 0.007 | 0.492 |
|  | adjusted for baseline score | 0.001 | n.a.* |
| Satisfaction with health | empty model | 0.015 | 0.574 |
|  | adjusted for baseline score | 0.002 | n.a.* |

# *Note:* * random effect variance = 0;

# Table S3: Results of Moran's test of the residuals from the multilevel models (final models)

| Statistics (Moran's I) | Observed rank | p-value (H0 = no autocorrelation) |
| --- | --- | --- |
| Affective well-being | | |
| I = 0,0306, | 868 | 0.264 |
| Life satisfaction | | |
| I = -0,0086 | 444 | 0.888 |
| Satisfaction with health | | |
| I = 0,0227 | 786 | 0.428 |

# *Note:* Moran's I calculated using Monte Carlo simulations with 1000 samples each using the *moran.mc* function from the R package *spdep*. Random permutations of a variable (the residuals) are repeatedly determined for the determined spatial weighting matrix and the rank of the observed I-value relative to the values of the simulations is considered.

# Table S4: Results of models with individual weights and principal component scores (PCA) instead of stringency index – for comparison

|  | Affective well-being | | Life satisfaction | | | Health satisfaction | | | |
| --- | --- | --- | --- | --- | --- | --- | --- | --- | --- |
|  | Stringency index weighted^1^ | PCA score (unweighted)^2^ | Stringency index weighted^1^ | PCA score (unweighted)^2^ | | Stringency index weighted^1^ | | PCA score (unweighted)^2^ | |
| Number of observations | N = 27,409 in 400 districts | | N = 29,464 in 401 districts | | | N = 29,871 in 401 districts | | | |
| **Fixed Effects:** | | | | |  | |  | |  |
| intercept | 1.598 (0.032)*** | 1.503 (0.0417)*** | 3.474 (0.095)*** | 3.327 (0.288)*** | | 3.813 (0.074)*** | | 3.824 (0.100)*** | |
| Initial pre-pandemic score | 0.550 (0.005)*** | 0.555 (0.005)*** | 0.535 (0.005)*** | 0.505 (0.005)*** | | 0.477 (0.005)*** | | 0.494 (0.005)*** | |
| Pandemic phase (ref.= 0) | | | | |  | |  | |  |
| 1 | -0.099 (0.018)*** | -0.090 (0.016)*** | -0.065 (0.040) | -0.050 (0.0380) | | 0.004 (0.048) | | -0.001 (0.045) | |
| 2 | -0.050 (0.026)^#^ | -0.028 (0.023) | -0.015 (0.061) | -0.025 (0.053) | | -0.078 (0.072) | | -0.080 (0.063) | |
| 3 | -0.019 (0.038) | 0.051 (0.039) | -0.040 (0.086) | 0.169 (0.080)* | | -0.117 (0.100) | | 0.033 (0.094) | |
| 4 | 0.181 (0.029)*** | 0.282 (0.059)*** | 0.054 (0.066) | 0.060 (0.113) | | 0.320 (0.077)*** | | 0.148 (0.149) | |
| 5 | 0.126 (0.029)*** | 0.254 (0.063)*** | -0.136 (0.065)* | -0.050 (0.137) | | 0.259 (0.076)*** | | 0.035 (0.156) | |
| 6a | 0.171 (0.030)*** | 0.305 (0.069)*** | -0.093 (0.066) | -0.008 (0.152) | | 0.113 (0.078) | | -0.107 (0.176) | |
| 6b | 0.078  (0.032)* | 0.230 (0.077)** | -0.112 (0.073) | -0.109 (0.170) | | -0.028 (0.081) | | -0.330 (0.199)^#^ | |
| 7 | -0.420 (0.100)*** | 0.154 (0.115) | -1.030 (0.233)*** | -0.331 (0.265) | | -0.102 (0.259) | | -0.462 (0.310) | |
| Medium stringency (increment 5) of previous NPIs | -0.004 (<0.001) | - | 0.001 (<0.001) | - | | 0.012 (<0.001) | | - | |
| PCA score (standardized) | - | -0.079 (0.030)** | - | 0.153 (0.222) | | - | | 0.095 (0.077) | |
| GISD score 2019 (0-1) | 0.017 (0.051) | 0.027 (0.024) | 0.159 (0.124) | 0.148 (0.058)* | | 0.095 (0.146) | | 0.137 (0.067)* | |
| ***Individual level covariates (pre-pandemic score)*** | | | | | | | | | |
| Disability | -0.051 (0.011)*** | -0.048 (0.012)*** | -0.131 (0.026)*** | -0.170 (0.028)*** | | -0.444 (0.031)*** | | -0.463 (0.033)*** | |
| Chronic illness | -0.062 (0.008)*** | -0.066 (0.008)*** | -0.122 (0.018)*** | -0.154 (0.018)*** | | -0.410 (0.022)*** | | -0.419 (0.022)*** | |
| Overweight | -0.002 (0.008) | -0.003 (0.008) | -0.021 (0.019) | 0.001 (0.019) | | -0.086 (0.022)*** | | -0.097 (0.022)*** | |
| Obesity | -0.030 (0.009)** | -0.026 (0.009)** | -0.089 (0.022)*** | -0.060 (0.022)** | | -0.297 (0.026)*** | | -0.322 (0.026)*** | |
| Age (per 5 years) | 0.001 (<0.001) | 0.006 (<0.001)*** | 0.008 (<0.001)** | -0.045 (<.0.001)*** | | -0.062 (<0.001)*** | | -0.055 (<0.001)*** | |
| Age^2^ (per 5^2^ years) | - | - | 0.003 (<0.001)*** | 0.003 (<.0.001)*** | | - | | - | |
| Gender (male) | 0.083 (0.007)*** | 0.092 (0.007)*** | -0.076 (0.016)*** | -0.058 (0.016)*** | | 0.048 (0.019)* | | 0.076 (0.019)*** | |
|  |  |  |  |  | |  | |  | |
| Education (CASMIN 3) (ref = low) | | |  |  | |  | |  | |
| medium | -0.002 (0.009) | 0.012 (0.009) | - | - | | - | | - | |
| high | 0.002 (0.010) | 0.025 (0.010)* | - | - | | - | | - | |
| Immigration history (Ref. = none) | | | | | | | | | |
| Parents/ second gen. | -0.010 (0.016) | 0.009 (0.015) | 0.053 (0.038) | 0.065 (0.037)^#^ | | 0.067 (0.044) | | 0.042 (0.043) | |
| Self /first gen. | -0.043 (0.010)*** | -0.033 (0.010)*** | 0.014 (0.024) | 0.093 (0.023)*** | | -0.002 (0.028) | | 0.108 (0.026)*** | |
| Log of net equivalent income | 0.040 (0.008)*** | 0.043 (0.007)*** | 0.160 (0.017)*** | 0.168 (0.016)*** | | 0.185 (0.020)*** | | 0.185 (0.019)*** | |
| Single parent | - | - | -0.091 (0.034)** | -0.085 (0.031)** | | - | | - | |
| Number of children up to 13 years | - | - | 0.032 (0.013)* | 0.043 (0.010)*** | | - | | - | |
| Married | - | - | 0.063 (0.019)*** | 0.102 (0.020)*** | | - | | - | |
| Person in need of care in household | - | - | -0.178 (0.040)*** | -0.197 (0.040)*** | | -0.128 (0.047)** | | -0.232 (0.046)*** | |
| *Control variables at district level* | | | | | | | | | |
| 7-day incidence (increment 10) | -0.005 (<0.001)*** | -0.007 (<0.001)*** | -0.006 (<0.001)*** | -0.003 (<0.001)** | | - | | - | |
| Cum. Number of vaccinations | -0.062 (0.018)*** | -0.052 (0.017)** | - | - | | - | | - | |
| Childcare rate for pre-school children | - | - | -0.002 (0.005) | -0.008 (0.003)*** | | - | | - | |
| **Interactions with stringency (PCA score)** | | | | | | | | | |
| Stringency x incidence | 0.001 (<0.001)** | <0.0001 (<0.001)*** | - | - | | - | | - | |
| Stringency/ PCA (+10) x disability | -0.007 (<0.001)* | -0.010 (0.012) | - | - | | - | | - | |
| Stringency/ PCA x chronic illness | 0.005 (<.0.001)* | 0.0195 (0.007)** | - | - | | - | | 0.079 (0.020)*** | |
| Stringency/ PCA x GISD | - | - | - | - | | -0.017 (<0.001) | | 0.134 (0.056)* | |
| Stringency x rate care preschool | - | - | -0.002 (<0.001)* | -0.002 (0.002) | | - | | - | |
| **Random effects (standard deviations)** | | | | | | | | | |
| districts | 0.142 | 0.029 | 0.345 | 0.083 | | 0.411 | | 0.096 | |
| error | 0.848 | 0.555 | 2.018 | 1.353 | | 2.382 | | 1.604 | |

*Note:* # p ≤ .10, * p ≤ .05, ** p ≤ .01, *** p ≤ .001; All continuous predictors were grand-mean centered; for binary variables, the non-occurrence is coded 0 as reference category;

^1^ same model with stringency index as predictor as in table 7 but with weighted cases;

^2^ same model as in table 7 but using the PCA score instead of the stringency index;
